# Computational fluid dynamics model and flow resistance characteristics of *Jatropha curcas* L xylem vessel

**DOI:** 10.1038/s41598-020-71576-9

**Published:** 2020-09-07

**Authors:** Tianyu Xu, Lixiang Zhang, Ze Li

**Affiliations:** Faculty of Civil Engineering and Mechanics, Kunming University of Science and Technology, Kunming, 650500 Yunnan China

**Keywords:** Computational biology and bioinformatics, Plant sciences

## Abstract

Xylem vessels are the channels used for water transport in *Jatropha curcas* L. Vessel complexity has a great influence on water transport. Therefore, using anatomical experiments and numerical simulations, the water transport characteristics of *J. curcas* L xylem vessels with perforation plate and secondary wall thickening (pit structures) were analyzed. The results showed that the xylem vessel in *J. curcas* provided a low resistance path. The xylem vessel resistance was composed of three elements: smooth vessels, secondary wall thickening and perforation plate. The proportion of smooth vessel resistance was the largest, accounting for 66.20% of the total resistance. Then the secondary wall thickening resistance accounted for 30.20% of the total resistance, and finally the perforation plate resistance accounted for 3.60% of the total resistance. The total resistance of the vessel model was positively correlated with the pit depth, perforation plate height and perforation plate width and negatively correlated with the vessel inner diameter and pit membrane permeability. The vessel inner diameter and the pit depth had a great influence on the total resistance. The total resistance of the vessel inner diameter of 52 µm was 89.15% higher than that of 61 µm, the total resistance of the pit depth of 5.6 µm was 21.98% higher than that of 2.6 µm. The pit structure in the secondary wall thickening caused the vessel to be transported radially, and the radial transmission efficiency of the vessel was positively correlated with the pit depth and pit membrane permeability and negatively correlated with the vessel inner diameter. The pit membrane permeability had the greatest influence on the radial transmission efficiency, and its radial transmission efficiency was 0–5.09%.

*Jatropha curcas* L trees are widely distributed in tropical and dry hot valleys^1^. Its seeds possess high oil content and can be easily converted into biodiesel. The lack of water resources has become one of the main limitations of *J. curcas* growth. Water transport from the roots to the leaves depends on the xylem^2,3^, while the water transport of xylem depends on the vessels^4,5^. During the growth and development of a xylem vessel, various shapes of wall thickening are formed, including annular thickening, helical thickening, reticulated thickening and pitted thickening^6,7^. The wall thickening structure has a great influence on the water transport of the xylem vessel^8,9^. In addition to the wall thickening structure of the vessel, there are several perforations on the end of the vessel, that is perforation plate^10^, and water must pass through these perforation plates during transport^11^.

In previous studies, water transport in plant xylem vessels was mainly used in botany observation experiments to explore flow characteristics^12^. Jeje^13^ measured the flow velocity of water in helical thickening vessels with different structures using a high-speed microscopy camera and analyzed the relationship between the helical thickening structure and the flow resistance of the vessel. Subsequently, Jeje^14^ created an enlarged secondary thickening model with plexiglass for experimental verification. The small size of the xylem vessels, the various types of structures and the complex internal flow phenomena lead to considerable difficulties for making botanical observations^2^. To compensate for the lack of experimental methods, some scholars have used computational fluid dynamics (CFD) methods to construct a fluid model of a plant’s xylem vessels, revealing the flow mechanism inside the xylem vessel. Roth^4^ established a two-dimensional simplified model of the vessel, analyzed the flow characteristics of the annular thickening, and concluded that the flow state was related to the distance and height between adjacent rings. Chen^8^ used an SST k–ε model to numerically simulate the annular thickening and helical thickening of the vessel. It was concluded that the vessel inner diameter and the thickening height had significant influence on the flow resistance and that the thickening width and inclination angle of thickening had less effects on the flow resistance. Ai QL^11^ simulated the pressure and velocity distribution of a scalariform perforation plate based on the k-ε model and found that the number of holes in the perforation plate was positively correlated with the total pressure drop.

These numerous experiments and numerical simulations obtain the conclusion that secondary wall thickening and perforation plates play important roles in xylem water transport. However, current studies have mainly focused on axial transmission within the vessel^8,9^. It has been observed under field emission scanning electron microscopy (FSEM)^15^ that the xylem vessels of *J. curcas* contain perforation plates and secondary wall thickening (pit structures) and that the presence of pit structures on the wall of the vessel result in radial water transport. In this paper, the method of combining anatomical experiments and numerical simulations was used to (1) obtain the resistance composition of different structures of the *J. curcas* vessel, (2) evaluate the total resistance of the change of the structural parameters of the vessel, and (3) analyze the radial transmission efficiency of the vessel. The results will provide a reference for the flow characteristics of the *J. curcas* vessel and obtain a deeper understanding of water transport in plant xylem.

## Methods

### Plant materials

In our study, trees (*J. curcas*) were collected by author at the Kunming University of Science and Technology in Yunnan, Southwest China (24° 84′ 50″ N, 102° 86′ 49″ E, 1,860 m above sea level) and were planted in a greenhouse by Faculty of Modern Agricultural Engineering. Our lab has identified plant materials used in research. The samples were deposited in Computer Key Laboratory of Kunming University of Science and Technology. Due to the samples are universal, no permit is required to collect such samples in part areas of Yunnan. The samples were taken from the 4-year-old *J. curcas* L. To ensure that the branches could be taken from the upper third of the tree crown, they were collected from heights of 1.5 m to 1.8 m. The samples were sliced within 3–4 days and analyzed by scanning electron microscopy.

### Slice preparation

#### Sawing the branches blocks

Taking the branches blocks of the *J. curcas* (0.7 cm × 0.7 cm × 0.7 cm), the cross section, tangential section and radial section were repaired under the dissecting microscope. The cross section was perpendicular to the longitudinal axis, and the tangential and radial sections were perpendicular to the wood ray.

#### Wood softening

The boiling method was used to soften the branches blocks of the *J. curcas*. The repaired blocks were put into a beaker filled with distilled water, and the beaker was vacuumed in a vacuum pump until the blocks sank to the bottom. The blocks were taken out and put into a pressure cooker for approximately 3–5 h until they were fully softened.

#### Slicing

The slicing knife was mounted on the microtome, tightened and the knife was slightly inclined. The degree of inclination depended on the hardness of the wood. The angle of the side of the experimental knife and the surface of the wooden block was approximately 10°. The thickness of the slice was generally 10 to 20 μm. After the sectioning was completed, the section was removed from the sectioning knife with a brush and transferred to a petri dish containing water. Three pieces of each side were cut for spares.

#### Packaging

The cover glass and glass slide were washed with alcohol before the cover, the slices were placed on the glass slide, and a layer of transparent agent was evenly applied on the slices (alcohol–glycerol 1:1). The cover glass was gently pressed onto the glass slide, and the bubbles were pushed out slowly.

#### Labeling

The label was pasted on the left side of the glass, which was then put it in the slice box for storage.

### FSEM observation and structure parameters

The sealed samples were soaked in distilled water and the glycerin was dislodged by multiple cleanings, then the samples were put into 30%, 50%, 70% and 90% ethanol solutions (30 min) to dislodge the moisture. Finally, the samples were put into a 100% ethanol solution for an hour, with metal spraying of the samples after air drying for at least 12 h. The samples were observed by using field emission scanning electron microscopy (FSEM) at the Analytic and Testing Research Center of Yunnan.

**Figure 1:**
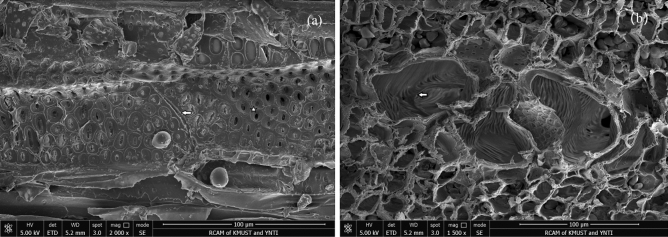
Field emission scanning electron microscopy of the *J. curcas* vessel (**a**) tangential section (**b**) cross section.

As observed from the micrographs of the tangential and cross sections, the xylem vessels contained perforation plate and secondary wall thickening (pit structures); the perforation plate had a single hole (arrow position), and the pit structure was a bordered pit (pentagram position). The tangential section and radial section of the sample were used to measure the parameters such as vessel length, pit diameter, pit aperture and perforation plate width (Fig. 1a). The cross section of the sample was used to observe the parameters such as inner diameter and perforation plate height (Fig. 1b). The detailed vessel structure parameters were used to analyze the water transfer characteristics. The dimensions and geometries of the xylem vessel are shown in Table 1.

**Table 1:** The patterns of the xylem vessel in the midribs of the branches of the *Jatropha.*

Structure parameters	*J. curcas*	Structure parameters	*J. curcas*
Cross section	Circular	Perforation plate width	3 μm
Vessel length	200 μm	Perforation plate height	3 μm
Inner diameter	55 μm	Pit diameter	8 μm
Pit type	Bordered	Pit aperture	2.6 μm
Perforation plate type	Single	Pit depth	4.6 μm
Perforation plate tilt angle	45°		

**Figure 2:**
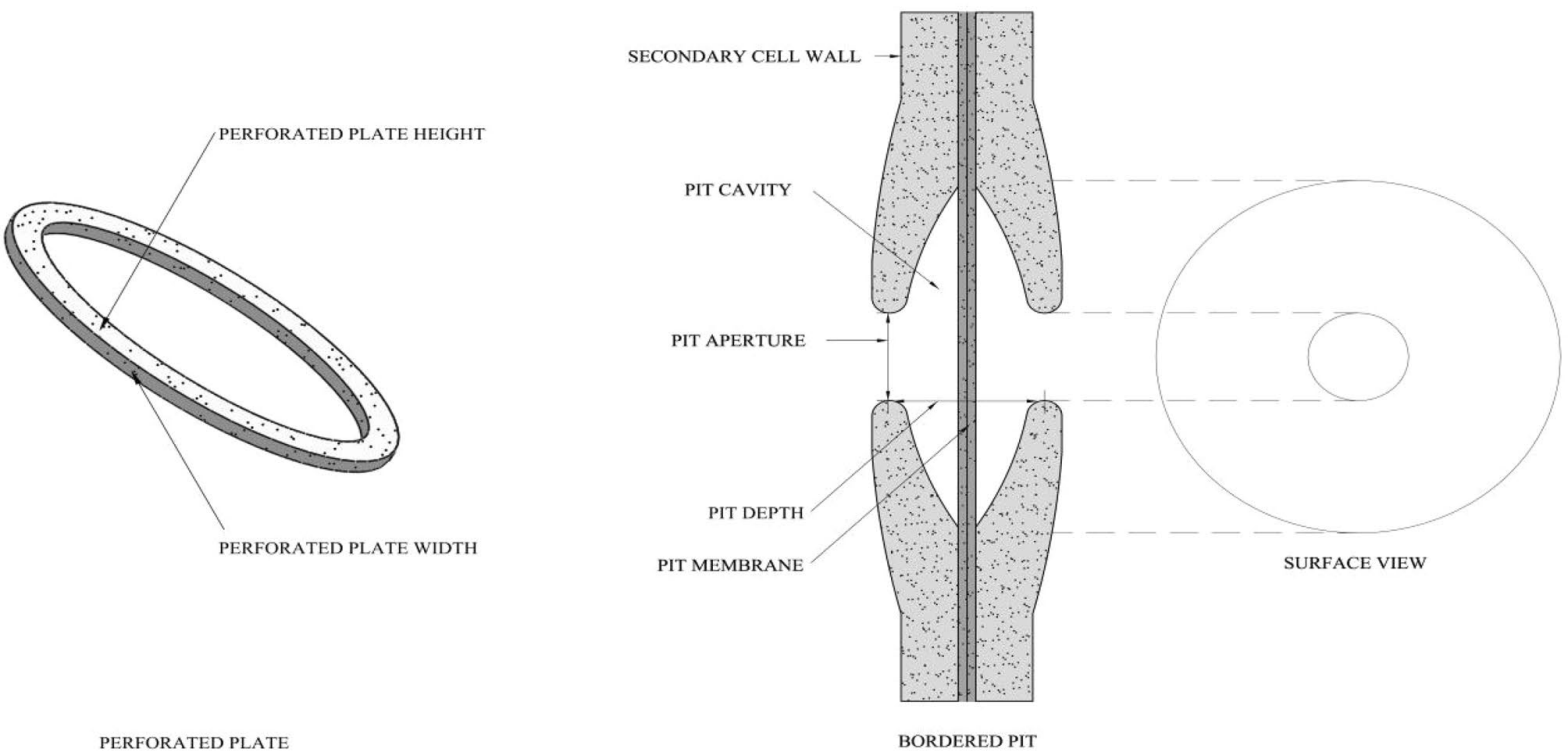
Schematic of the pit structure and perforation plate.

In our study, secondary wall thickening was characterized as pitted thickening, which was part of the nonthickening hole or depression left by the secondary wall during the thickening process. Figure 2 shows the elements of the vessel structure.

### Modeling approaches

**Figure 3:**
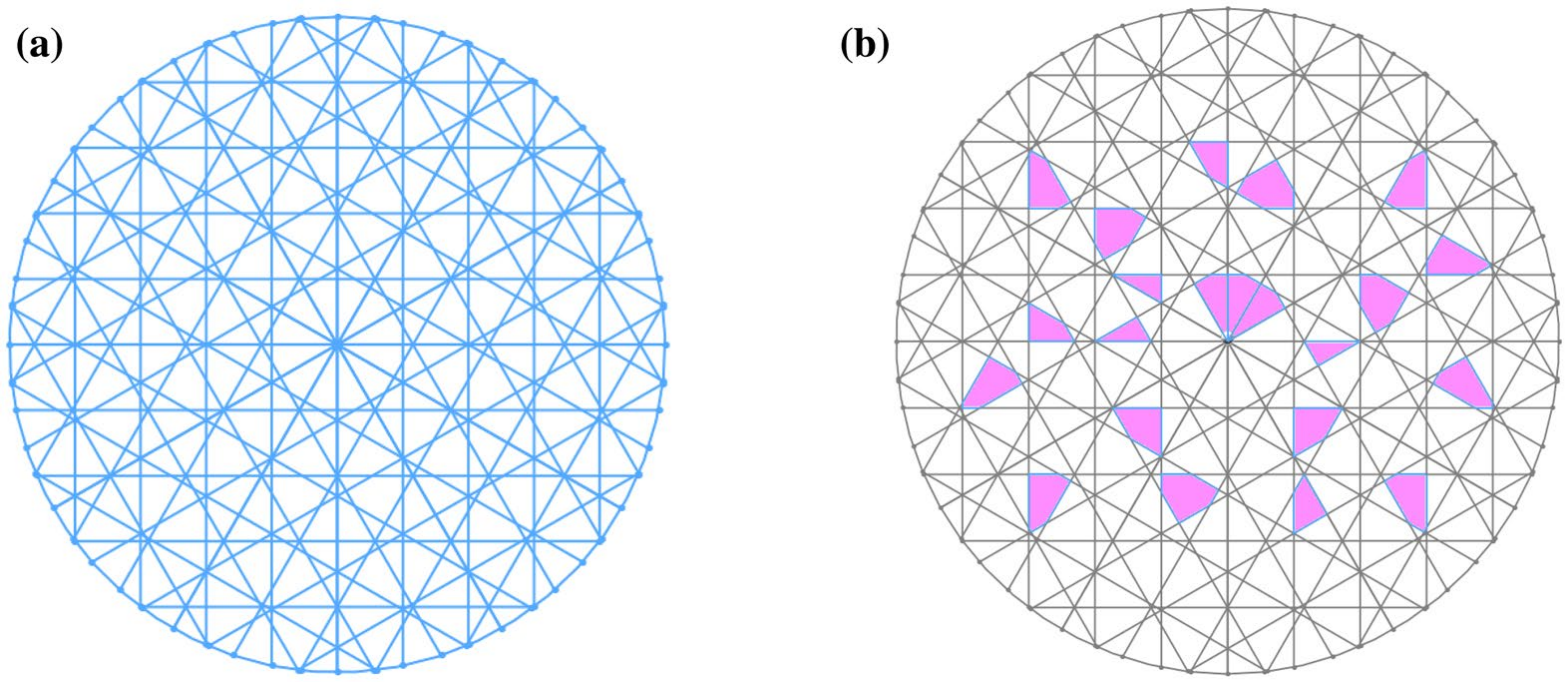
Schematic diagram of pit membrane (**a**) Pit membrane structure (**b**) porosity distribution.

A complete three-dimensional model of the vessel was established based in SolidWorks. The pit membrane structure in the model was important, as it behaved as a safety valve for water transport in plants. In this section, combining the anatomical observation and mathematical model, and considering the permeability of the pit membrane, the expression of the pit membrane was carried according to the literature^16,17^ and the structure is shown in Fig. 3a. In our study, the pit membrane permeability was 15% by anatomical observation(Fig. 3b).

**Figure 4:**
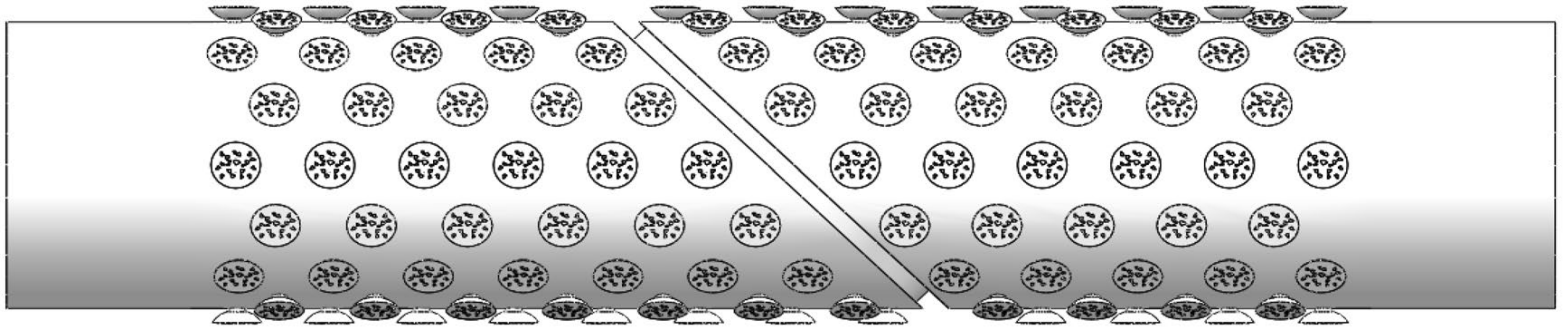
The 3D models of the vessel.

Therefore, 3D models with perforation plate and secondary wall thickening were built, as shown in Fig. 4, where the thickness of the pit membrane was 0.2 μm.

### Calculation method and initial condition

In a general approach, the fluid flow relies on the steady-state conservation equations for mass and momentum in a fluid, which are given by:

Continuity equation:

1 $$ \frac{\partial u}{{\partial x}} + \frac{\partial v}{{\partial y}} + \frac{\partial w}{{\partial z}} = 0 $$

Momentum equation:

2 $$ \left\{ {\begin{array}{*{20}c} {\rho \left( {u\frac{\partial u}{{\partial x}} + v\frac{\partial u}{{\partial y}} + w\frac{\partial u}{{\partial z}}} \right) = - \frac{\partial P}{{\partial x}} + \mu \left( {\frac{{\partial^{2} u}}{{\partial x^{2} }} + \frac{{\partial^{2} u}}{{\partial y^{2} }} + \frac{{\partial^{2} u}}{{\partial z^{2} }}} \right)} \\ {\rho \left( {u\frac{\partial v}{{\partial x}} + v\frac{\partial v}{{\partial y}} + w\frac{\partial v}{{\partial z}}} \right) = - \frac{\partial P}{{\partial y}} + \mu \left( {\frac{{\partial^{2} v}}{{\partial x^{2} }} + \frac{{\partial^{2} v}}{{\partial y^{2} }} + \frac{{\partial^{2} v}}{{\partial z^{2} }}} \right)} \\ {\rho \left( {u\frac{\partial w}{{\partial x}} + v\frac{\partial w}{{\partial y}} + w\frac{\partial w}{{\partial z}}} \right) = - \frac{\partial P}{{\partial z}} + \mu \left( {\frac{{\partial^{2} w}}{{\partial x^{2} }} + \frac{{\partial^{2} w}}{{\partial y^{2} }} + \frac{{\partial^{2} w}}{{\partial z^{2} }}} \right)} \\ \end{array} } \right. $$

where *u*, *v*, *w* are the components of the velocity vector along the *x*, *y*, *z*-directions, respectively, *ρ* is the fluid density, *P* is the fluid pressure, *µ* is the dynamic viscosity.

**Figure 5:**
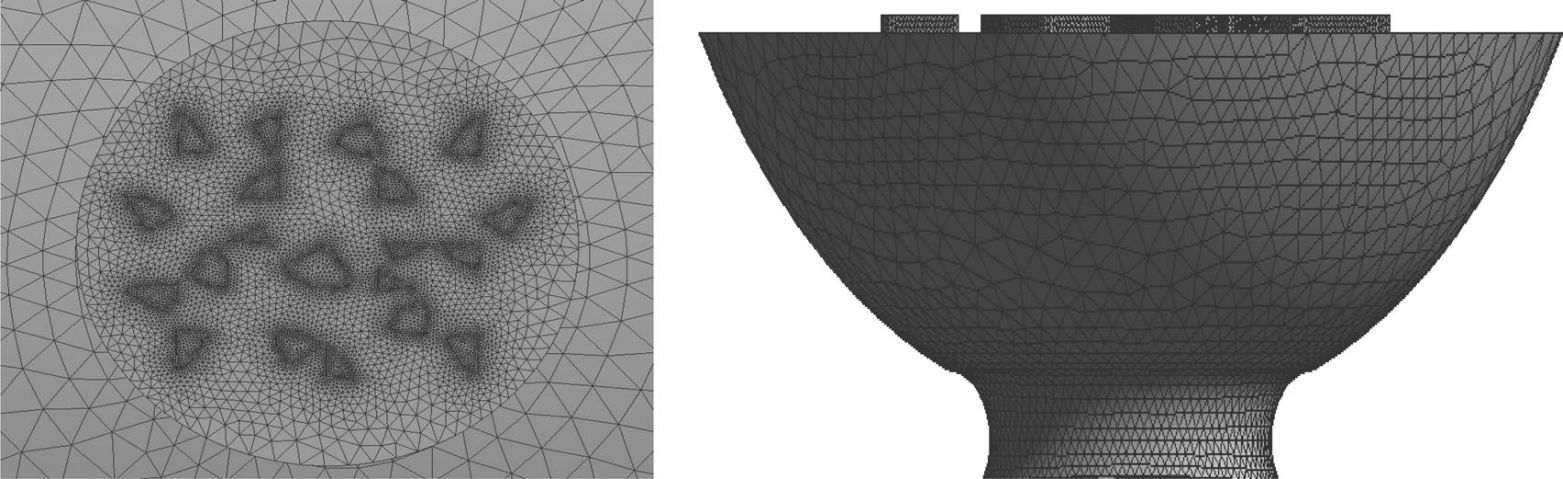
Grids in part of vessel model.

The simulation of the model was a fluid domain, and the fluid was water. The SIMPLEC scheme was chosen to achieve the coupling solution for the pressure and velocity equations. For the boundary conditions, the pressure was zero at the model outlet, and the flow velocity was 0.3 mm s^−1^ at the model inlet. To avoid the effects of the entrance and exit, an extended smooth segment with a length of 25 μm was added at both ends of the microchannels. Considering the irregularity of the vessel, grid generation was performed using tetrahedral and hexahedral nongrid structures. Based on the prediction accuracy of the inlet and outlet pressure drop, a grid independence test was performed. The predicted pressure drop difference was within 0.5%, the number of grids was considered to have no effect on the results. The total number of grids in the model was 10 million. The maximum and minimum unit sizes were 7.64 × 10^–6^ m and 3.82 × 10^–8^ m, respectively. PowerCube-S01 with a high-performance computing system was used for the simulation. Figure 5 shows the local mesh of the pit structure.

## Results

### Flow velocity and pressure gradient on the vessel

**Figure 6:**
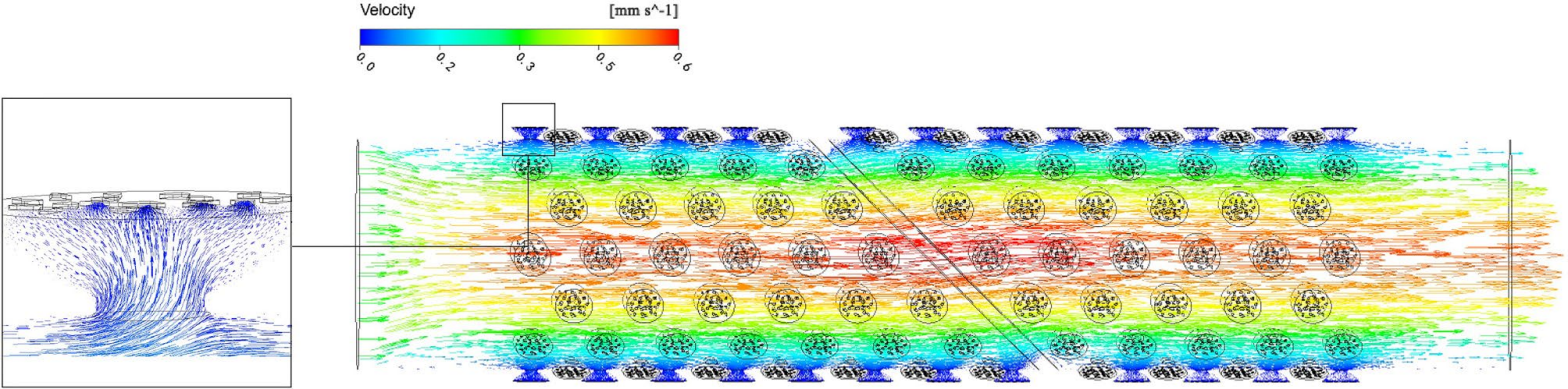
Velocity distribution of flow field in section along vessel axis.

**Figure 7:**
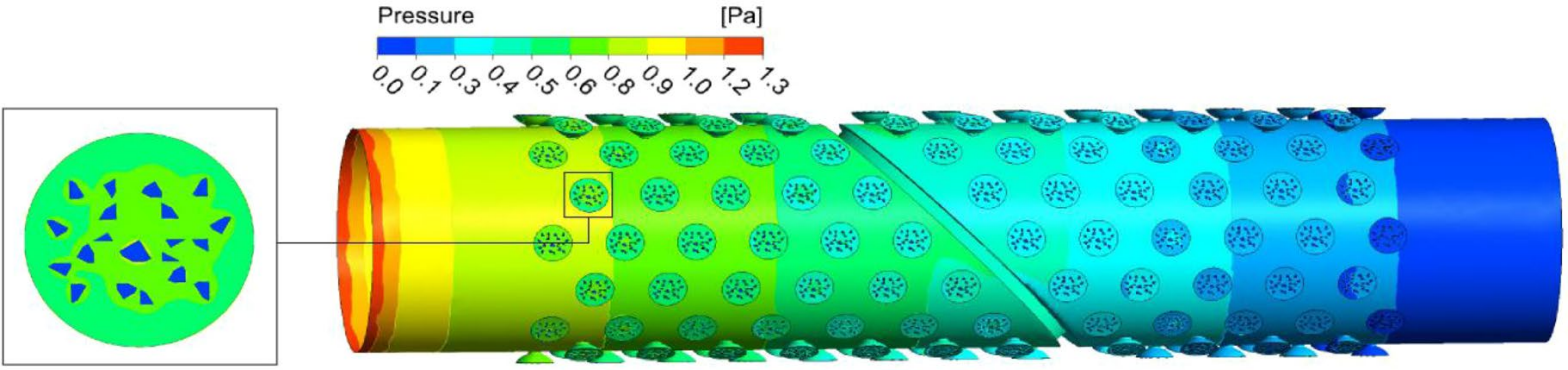
Pressure distribution of flow field in section along vessel axis.

The fluid velocity and pressure at all points in the model were not the absolute flow velocities in the *J. curcas* vessel, under any particular flow state, occurring in any whole plant in the greenhouse (Figs. 6, 7). However, the compared velocities within different components (perforation plate and secondary wall thickening) were valid. Within an individual vessel, the maximum velocity was intensely focused on the perforated plate area. For the pit structure, the radial flow was clearly visible and converged at the outflow point on the pit membrane, and the fluid in the pit cavity did not take part in the vessel axial flow. In the model, the general flow velocity inside the pit cavity was extremely low, and the pit structure had a great influence on the flow state in the pit cavity. The pressure gradient of the vessel wall is shown in Fig. 7, which also shows the local pressure gradient of the pit structure. The pressure distribution gradient of the pit membrane and perforated plate region was complicated. At the inlet and outlet regions of the vessel, the pressure gradient was less steep up to the perforated plate. However, directly before the secondary wall thickening, a small region of higher pressure developed, and an area of low pressure was present after the perforated plate. In the pit structure area, the pressure on the pit membrane was lower than that on the surrounding vessel area, while the pressure on the pores surrounding the area was higher than that on the other areas of the pit membrane.

### Components of total resistance: perforation plate, pit structures, and smooth vessel

To estimate the different structures of the *J. curcas* vessel components, the perforation plate, secondary wall thickening (bordered pit) and smooth vessel, the total pressure (*Δp*) and the average flow rate (*q*) were obtained through numerical simulation. The total resistance of the *J. curcas* vessel was calculated from *Δp* divided by *q*. In the initial calculation, the smooth vessel was added to model 1, secondary wall thickening was added to model 2, and the perforation plate was added to model 3.

With the addition of secondary wall thickening in model 1 (Table 2), *Δp* increased by 20.26%, the flow rate decreased by 21.08%, and the flow resistance increased by 45.61%. In models 2 and 3, *Δp* increased by 3.72% with the addition of the perforation plate, *q* was constant, and the flow resistance increased by 3.72%.

**Table 2:** Flow resistance of the vessel model.

Models	Δp/Pa	q/(m^3^ s^−1^)	Flow resistance/(Pa s m^−3^)
Model 1	0.849	7.093 × 10^–13^	1.197 × 10^12^
Model 2	1.021	5.858 × 10^–13^	1.743 × 10^12^
Model 3	1.059	5.858 × 10^–13^	1.808 × 10^12^

**Figure 8:**
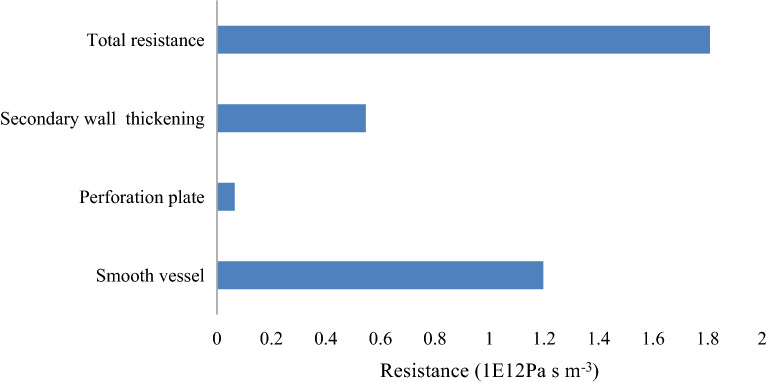
Total resistance component of *J. curcas* vessel model.

As shown in Table 3, the resistance ratio of each component in the vessel was obtained. The results are shown in Fig. 8. The perforation plate was a relatively lower component, at 3.60% of the total resistance; secondary wall thickening accounted for 30.20% of the total resistance, and the smooth vessel was a relatively larger component, at 66.20% of the total resistance.

**Table 3:** Resistance calculation method.

Calculation method	Formula	Calculation method	Formula
Smooth vessel flow resistance	R_s_ = R_1_	Fraction due to smooth vessel	F_1_ = R_1_/R_3_
Secondary wall thickening flow resistance	R_w_ = R_2_ − R_1_	Fraction due to secondary wall thickening	F_2_ = R_2_ − R_1_/R_3_
Perforation plate flow resistance	R_p_ = R_3_ − R_2_	Fraction due to perforation plate	F_3_ = R_3_ − R_2_/R_3_
Total flow resistance	R_tot_ = R_3_		

R_1_ is the flow resistance of the model 1; R_2_ is the flow resistance of the model 2; R_3_ is the flow resistance of the model 3.

### Effects of the changes in the vessel structural parameters on total resistance

#### Effect of pit membrane permeability on total resistance

**Figure 9:**
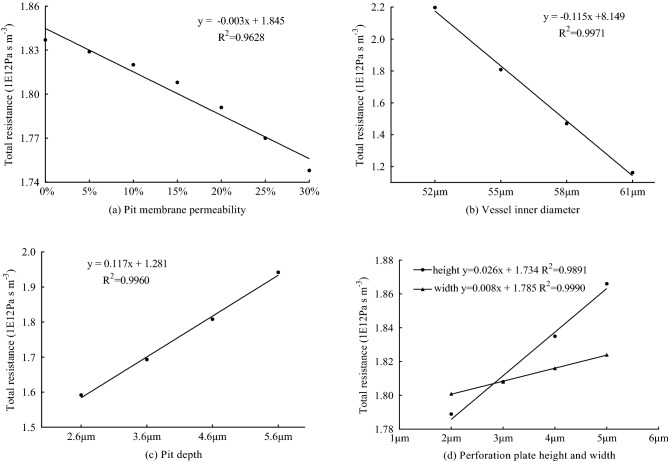
Influence of vessel model parameters on total resistance: (**a**) pit membrane permeability, (**b**) vessel inner diameter, (**c**) pit depth, (**d**) perforation plate height and width.

The radial water transport of the vessel relied on the pit structure, and the pit membrane permeability in the pit had a significant effect on water transport. There was a close linear relationship between the flow resistance and pit membrane permeability (Fig. 9a), which was notably affected by the pit membrane permeability. When the pit membrane permeability changed from 0 to 30%, the total flow resistance changed from 1.837 × 10^12^ to 1.748 × 10^12^ Pa s m^−3^. The flow resistance of the vessel decreased with increasing pit membrane permeability. In the *J. curcas* vessel, the flow resistance of the pit membrane permeability of 30% was 5.09% higher than that of 0%.

#### Effect of vessel inner diameter on total resistance

The flow rate and total pressure of the vessel were affected the size of the vessel inner diameter and then affected the flow resistance of the vessel. There was a close linear relationship between the flow resistance and the vessel inner diameter (Fig. 9b), which was notably affected by the vessel inner diameter. When the vessel inner diameter changed from 52 to 61 µm, the total flow resistance changed from 2.198 × 10^12^ to 1.162 × 10^12^ Pa s m^−3^. The flow resistance of the vessel decreased with increasing vessel inner diameter. In the *J. curcas* vessel, the flow resistance of the vessel inner diameter of 52 µm was 89.15% higher than that of 61 µm.

#### Effect of pit depth on total resistance

The vessel inner diameter was affected by the size of the pit depth and then affected the flow, *Δp* and flow resistance of the vessel. There was a close linear relationship between the flow resistance and the pit depth (Fig. 9c), which was notably affected by the pit depth. When the pit depth changed from 2.6 to 5.6 µm, the total flow resistance changed from 1.592 × 10^12^ to 1.942 × 10^12^ Pa s m^−3^. The flow resistance of the vessel increased with increasing pit depth. In the *J. curcas* vessel, the flow resistance of the pit depth of 5.6 µm was 21.98% higher than that of 2.6 µm.

#### Effect of perforation plate height and perforation plate width on total resistance

The flow velocity in the vessel was affected by the size of the perforation plate and then affected by the flow resistance of the vessel. There was a close linear relationship between the flow resistance and the perforation plate (Fig. 9d), which was notably affected by the perforation plate. When the perforation plate height changed from 2 to 5 µm, the total flow resistance changed from 1.789 × 10^12^ to 1.866 × 10^12^ Pa s m^−3^, and when the perforation plate width changed from 2 to 5 µm, the total flow resistance changed from 1.801 × 10^12^ to 1.824 × 10^12^ Pa s m^−3^. The flow resistance of the vessel increased with increasing perforation plate height and perforation plate width. In the *J. curcas* vessel, the flow resistance of the perforation plate height of 5 µm was 3.78% higher than that of 2 µm, and the flow resistance of the perforation plate width of 5 µm was 1.28% higher than that of 2 µm.

### Radial transmission efficiency of the vessel

To study the effect of the radial transport of the vessel on the total resistance, the vessel inner diameter, pit depth and pit membrane permeability were analyzed. Here, the radial transmission efficiency is expressed as the percentage of radial transmission resistance in the total resistance

3 $$ F = \left( {F_{2} - F_{1} } \right)/F_{1} $$

where *F* is radial transmission efficiency, *F*_2_ is the flow resistance with only axial water transport (The pit membrane permeability is 0), *F*_1_ is the flow resistance with axial and radial water transport.

**Figure 10:**
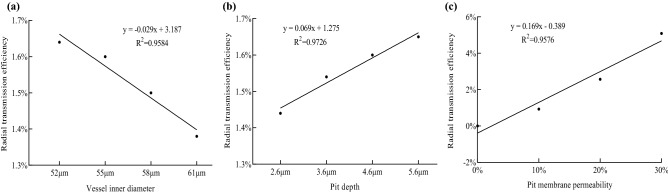
Influence of vessel model parameters on radial transmission efficiency: (**a**) vessel inner diameter, (**b**) pit depth, (**c**) pit membrane permeability.

The influence of different structural parameters on *F*_1_ and *F*_2_ is shown in Table 4. The radial transmission efficiency of the *J. curcas* vessel was calculated by Eq. (1). The results show that the radial transmission efficiency of the vessel had a good correlation with the vessel inner diameter, pit depth and pit membrane permeability (Fig. 10). When the vessel inner diameter changed from 52 to 61 µm, the radial transmission efficiency changed from 1.64 to 1.38%; when the pit depth changed from 2.6 to 5.6 µm, the radial transmission efficiency changed from 1.44 to 1.65%; and when the pit membrane permeability changed from 0 to 30%, the radial transmission efficiency changed from 0 to 5.09%. Under the other geometrical dimensions, the radial transmission efficiency of the vessel decreased with increasing vessel inner diameter and increased with increasing pit depth and pit membrane permeability.

**Table 4:** Flow resistance of vessel with different structural parameters.

Vessel inner diameter (µm)	F_1_ (× 10^12^)	F_2_ (× 10^12^)	Pit depth (µm)	F_1_ (× 10^12^)	F_2_ (× 10^12^)	Pit membrane permeability (%)	F_1_ (× 10^12^)	F_2_ (× 10^12^)
52	2.198	2.234	2.6	1.592	1.615	0	1.837	1.837
55	1.808	1.837	3.6	1.693	1.719	10	1.820	1.837
58	1.470	1.492	4.6	1.808	1.837	20	1.791	1.837
61	1.162	1.178	5.6	1.942	1.974	30	1.748	1.837

## Discussion

Based on the modeling of botanical anatomical vessels, the resistance distribution of each structure in the vessel and its influence on the vessel were analyzed. The radial transmission efficiency was obtained by comparing it with the vessel that using axial transmission. The maximum flow velocity in the vessel occurred in the area of the perforated plate. The reason is that the perforation plate decreased the inner diameter of the vessel. Ai QL^11^ analyzed the perforation plate and obtained a similar flow velocity distribution. The secondary wall thickening structure showed a less steep pressure gradient than that of the perforation plate. It may be that the pit cavity was outside the vessel inner diameter and that the energy loss generated by the low velocity flow inside the pit cavity was insufficient to exceed the pressure gradient. On the other hand, the perforation plate on the inside of the vessel wall reduced the inner diameter and increased the velocity of the fluid^8^, therefore, the pressure gradient changed greatly. Although the flow velocity inside the pit cavity was very low, it had a great influence on a small pit cavity, causing the pressure of the pit membrane to larger charge.

The xylem vessel model of *J. curcas* showed the flow phenomena of different resistances in xylem vessels, including perforation plate, secondary wall thickening and smooth vessels. The components of the resistance of the three elements showed that the smooth vessel obtained the largest flow resistance, indicating that the vessel was close to the ideal smooth vessel and that water transport was more efficient, which also explained the evolution of the plant^12^. The secondary wall thickening resistance was second, increasing the inner wall thickness to stabilize the vessel and the water radial flow. Schulte^18^ analyzed the resistance of a 5-hole perforation plate and found that the resistance was only 8% of the total resistance. In our study, the perforation plate accounted for 3.60% of the total resistance, which had the lowest flow resistance in the water transport. The main reason was that the perforation plate was a single hole, but the perforation plate structure made the vessel more stable mechanically^5^.

The change in the vessel inner diameter had an important effect on the total resistance of the vessel. This was mainly because that as the vessel inner diameter increased, the flow rate inside the vessel increased, the pressure drop decreased, and the total resistance decreased. Similar trends were found by Tyree^6^ and Chen^8^, who showed that in a larger inner diameter vessel, the water transport efficiency was much closer to that of an ideal vessel because the structural features of the vessel changes had little effect. Pit depth was also an important parameter that affected the total resistance. Because the distance between the pit aperture and the pit membrane was affected, the vessel inner diameter was changed^19,20^. Compared with the perforation plate width, the perforation plate height had a greater influence on the flow resistance. The perforation plate height had a large influence on the fluid velocity in the vessel, resulting in a large change in the total resistance^11^.

At present, the study of xylem vessels is based on the influence of the internal structure on axial transmission^9^. In our study, the area of secondary wall thickening (pit structures) led to radial flow inside the pit cavity, indicating that water could be transferred between vessels^20^. From the analysis of the vessel inner diameter, pit depth and pit membrane permeability of the vessel, the pit membrane permeability had the greatest influence on its radial transmission efficiency with the transmission efficiency of 0–5.09%, indicating that the vessel was important for axial transmission. The radial transmission efficiency of the vessel was positively correlated with the pit depth and pit membrane permeability and negatively correlated with the vessel inner diameter. The decrease in the pit depth led to an increase in the curvature of the inner wall of the pit cavity, and the sudden expansion of the fluid caused high shear forces in the fluid, which was not conducive to water transport^21^. The increase in the vessel inner diameter increased the flow rate in the vessel, which increased the axial transmission capacity, so the radial transmission capacity was reduced. As the pit membrane permeability increased, the flow rate in the pit cavity increased, the pressure drop decreased, and the radial transmission capacity increased^22^. The results of this study are contribute to the further study of the role of various structures in vessels.

## Conclusions

The xylem vessel in *J. curcas* provides a low resistance path, in which the smooth vessel (resistance along the path) has the largest resistance, followed by secondary wall thickening and the perforation plate. Model solutions demonstrated a close positive relationship between the total resistance of vessels with pit depth, perforation plate height and width and a negative relationship between the total resistance of vessels with the vessel inner diameter and pit membrane permeability. Among them, pit depth and the vessel inner diameter had a great influence on the total resistance. The radial transmission efficiency of the vessel was positively correlated with the pit depth and pit membrane permeability and negatively correlated with the vessel inner diameter. Meanwhile, pit membrane permeability had a great influence on the radial transmission efficiency.

## Data Availability

The data that support the findings of this study are available from the corresponding author on reasonable request.
